# Metastasis of Invasive Ductal Carcinoma of the Breast to the Uterine Cervix 30 Years Post‐Mastectomy: A Case Report

**DOI:** 10.1111/jog.70509

**Published:** 2026-09-28

**Authors:** Taro Miyake, Hidenori Sasa, Tsubasa Ito, Takanori Watanabe, Takako Sato, Sho Ogata, Masashi Takano

**Affiliations:** ^1^ Department of Obstetrics and Gynecology National Defense Medical College Hospital Saitama Japan; ^2^ Department of Laboratory Medicine National Defense Medical College Hospital Saitama Japan

**Keywords:** breast cancer, HER2‐positive, invasive ductal carcinoma, metastasis, uterine cervix

## Abstract

Metastasis of invasive ductal carcinoma of the breast to the uterine cervix is rare, with only one report of a case occurring more than 20 years after the primary surgery. A 69‐year‐old woman presented with abnormal uterine cervical cytology (atypical glandular cells without tumor diathesis) 30 years after undergoing breast cancer surgery. Magnetic resonance imaging revealed a 38‐mm cervical mass. Modified radical hysterectomy was performed. The resected specimen exhibited an immunohistochemical profile of GATA3(+)/mammaglobin(+)/PAX8(−), which definitively excluded primary cervical adenocarcinoma and confirmed the diagnosis of metastatic invasive ductal carcinoma. Crucially, although the primary tumor was hormone receptor‐positive, cervical metastasis exhibited receptor conversion to HER2‐positive status. This case underscores the importance of considering metastatic breast cancer in the differential diagnosis of cervical masses in breast cancer survivors regardless of the disease‐free interval. This further highlights the necessity for receptor re‐evaluation in metastatic lesions to guide appropriate targeted therapy.

## 1 Introduction

Breast cancer frequently metastasizes to the bones, lungs, liver, and brain; however, metastasis to gynecological organs is uncommon. Although invasive ductal carcinoma (IDC) accounts for most invasive breast cancers, invasive lobular carcinoma (ILC) is overrepresented in cases of metastasis to gynecological organs, including the uterus and cervix. Therefore, uterine cervical metastasis from IDC is rare [1]. Detection of extragenital malignancies via cervicovaginal cytology is rare. According to a literature review, breast cancer accounts for a minority of extrauterine malignancies detected using Papanicolaou test [2]. To date, approximately 35–40 cases of breast cancer with metastasis to the uterine cervix have been reported [3]. Histologically, ILC occurs more frequently than IDC [1]. In a review of 26 reported cases, solitary metastasis to the cervix was identified in only two cases, with the majority presenting as part of a systemic disseminated disease [4]. Although the risk of breast cancer recurrence is highest in the early years after surgery, it can persist long‐term [5, 6]. A case of cervical metastasis from recurrent IDC more than 20 years after the initial diagnosis has been reported [3]; however, recurrence approximately 30 years after the primary surgery is exceptional. Herein, we report a rare case of IDC metastasizing to the uterine cervix 30 years after the primary surgery that was diagnosed via cervical cytology and confirmed by immunohistochemistry.

## 2 Case Presentation

A 69‐year‐old woman (gravida 4, para 3) was referred to our hospital after uterine cervical cytology revealed adenocarcinoma. She was diagnosed with left breast cancer at the age of 39 years and underwent a modified radical mastectomy (Patey procedure). The primary tumor was estrogen receptor (ER)–positive, progesterone receptor (PgR)–positive, and human epidermal growth factor receptor 2 (HER2)–negative. Postoperatively, the patient received six cycles of chemotherapy (mitomycin C, cyclophosphamide, methotrexate, and 5‐fluorouracil). At 44 years old, the patient developed recurrence in the left supraclavicular lymph nodes and chest wall. She was treated with doxifluridine and cyclophosphamide and achieved complete remission at the age of 48 years, followed by maintenance therapy with tamoxifen. At the age of 66 years, recurrence in the mediastinal lymph nodes caused esophageal stenosis and dysphagia. Gastrostomy was performed, and the patient was maintained on oral capecitabine and cyclophosphamide.

Routine cervical cancer screening at 69 years of age revealed adenocarcinoma. Subsequent cervical and endometrial cytology performed at our institution (Figure 1) confirmed the presence of numerous atypical glandular cells, both in overlapping clusters and as isolated cells. These cells were monomorphic. Although round‐to‐oval nuclear enlargement was noted, the nuclear atypia was mild. Notably, the background was clean and lacked tumor diathesis, and intracytoplasmic lumina (ICL) were identified. Tumor markers showed elevated carcinoembryonic antigen (30.8 ng/mL), whereas cancer antigen 125, carbohydrate antigen 19‐9, and squamous cell carcinoma levels were within normal limits. Pelvic magnetic resonance imaging revealed an irregular solid mass measuring approximately 38 mm in the uterine cervix, with suspicion of parametrial invasion. Colposcopy‐directed biopsy demonstrated round tumor cells infiltrating the cords and small nests, which were positive for GATA binding protein 3 (GATA3) and mammaglobin but negative for paired box gene 8 (PAX8) and p16INK4a, encoded by *CDKN2A* (p16).

**FIGURE 1:**
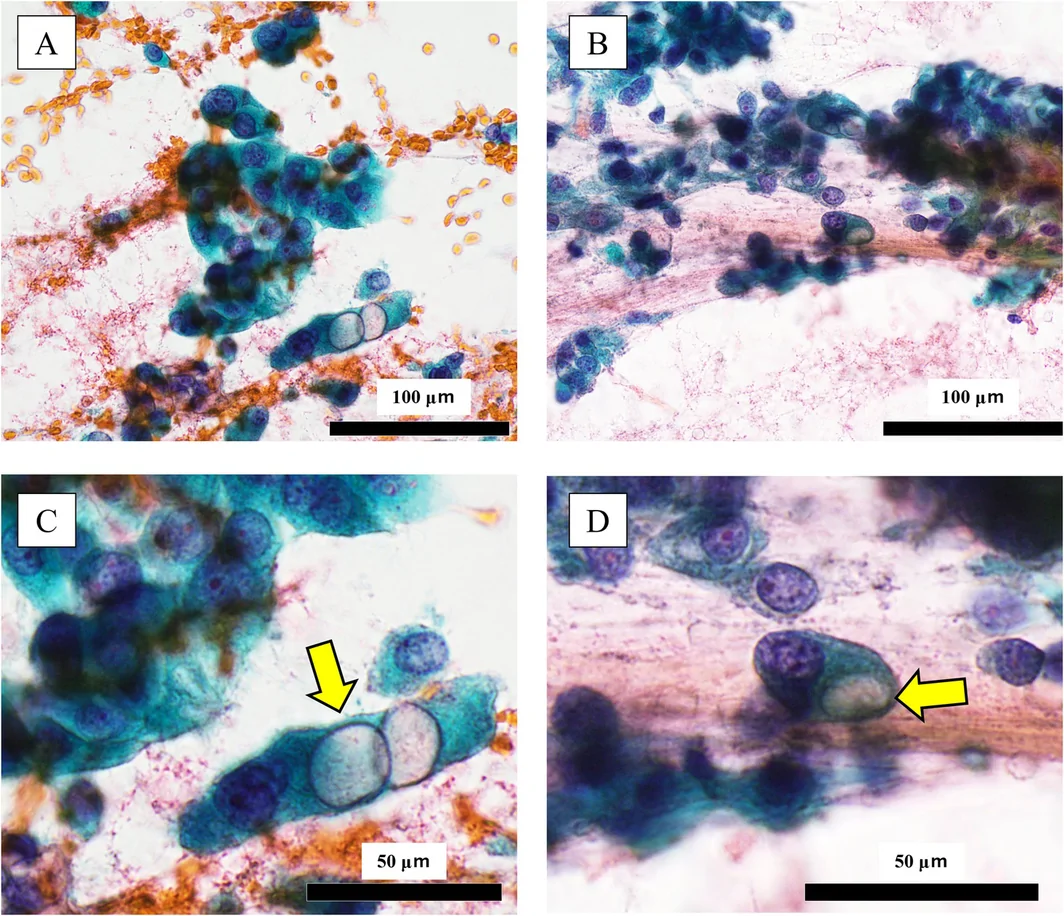
Cytological findings of uterine cervical and endometrial smears Papanicolaou stain; original magnification, ×60 objective. (A, C) Endometrial smear. (B, D) Cervical smear. The background was clean and lacked tumor diathesis typically associated with primary cervical adenocarcinoma. The cells appeared as overlapping clusters of atypical glandular cells. (B, D) Although nuclear atypia was mild, with a relatively monomorphic appearance, the cells exhibited a high nuclear‐to‐cytoplasmic ratio and intracytoplasmic lumina (arrows). (C) Similar intracytoplasmic lumina were also observed in endometrial cells (arrow).

Based on the patient's history of breast cancer, the cytological features that were atypical for a primary cervical adenocarcinoma, and the immunohistochemical profile of the colposcopy‐directed biopsy, cervical metastasis of breast cancer was strongly suspected. Nonetheless, modified radical hysterectomy and bilateral salpingo‐oophorectomy were performed for a definitive diagnosis and local control. Macroscopically, the resected specimen contained a 28 × 26 × 26‐mm yellow‐white solid mass in the cervix. Histologically, the cervical tumor cells exhibited infiltration in the cords, small nests, and occasional tubules, which are features consistent with metastatic IDC. No tumor cells were found in the uterine corpus or ovaries. Multiple pelvic lymph nodes tested positive for metastasis. The immunohistochemical profile was cytokeratin 7(+), cytokeratin 20(−), GATA3(+), gross cystic disease fluid protein‐15 (GCDFP‐15)(+), mammaglobin(+), PAX8(−), and p16(focal +). The biomarker status was ER(−)/PgR(−) and HER2 (3+). Because the original pathological slides of the primary breast tumor were unavailable, a pathological review of the metastatic lymph node specimen obtained at the time of the primary surgery was performed at our institution. This review revealed features consistent with ductal carcinoma, showing morphological similarities to uterine cervical metastasis, despite biomarker discordance. The clinicopathological and immunohistochemical findings of the three specimens are summarized in Table 1, and representative histological findings are shown in Figure 2. Based on the HER2‐positive status, the patient was switched to targeted therapy (trastuzumab and pertuzumab) combined with oral doxifluridine and cyclophosphamide. The patient has been alive and well for more than 18 months after the surgery, with ongoing outpatient therapy.

**TABLE 1:** Comparison of pathological and immunohistochemical findings among the three specimens.

Variable	LN biopsy at primary breast cancer (39 year)	Cervical biopsy at recurrence (69 year)	Hysterectomy specimen (69 year)
Diagnosis	Metastatic carcinoma, suggestive of breast origin	Adenocarcinoma	Metastatic breast carcinoma
ER	Strong positive	N/A	Negative
PgR	Strong positive	N/A	Negative
HER2	Negative	N/A	3+
Ki‐67	Few positive cells	N/A	N/A
GATA3	N/A	Positive	Positive
Mammaglobin	N/A	Partially positive	Partially positive
GCDFP‐15	N/A	N/A	Positive
PAX8	N/A	Negative	Negative
p16	N/A	Negative	Focal positive
CK7	N/A	N/A	Positive
CK20	N/A	N/A	Negative
Overall interpretation	Breast origin supported	Breast recurrence/metastasis suspected	Consistent with metastatic breast carcinoma

*Note:* The three tissue specimens include metastatic lymph nodes obtained during primary breast cancer surgery (age: 39 years), cervical biopsy at the time of recurrence (age: 69 years), and definitive hysterectomy specimen (age: 69 years). A notable conversion from hormone receptor‐positive to HER2‐positive status was observed between the initial and recurrent lesions.

Abbreviations: CK, cytokeratin; ER, estrogen receptor; GCDFP‐15, gross cystic disease fluid protein‐15; HER2, human epidermal growth factor receptor 2; LN, lymph node; N/A, not available; PgR, progesterone receptor.

**FIGURE 2:**
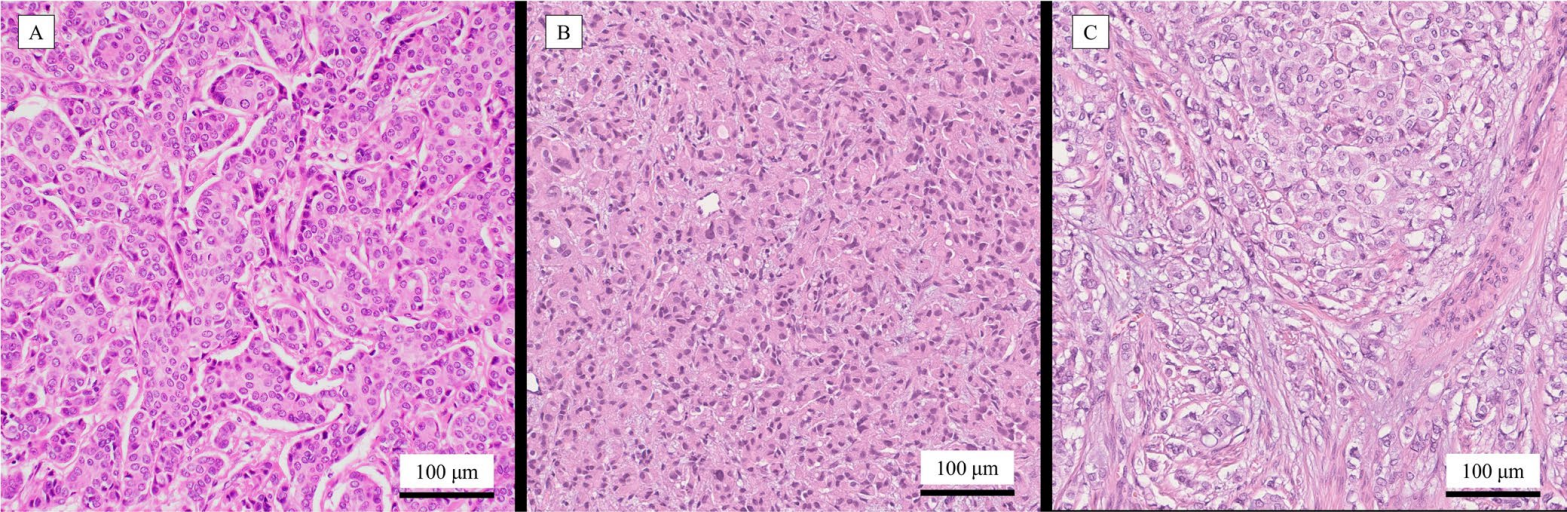
Histopathological findings of the primary breast cancer and uterine cervical metastatic lesions (hematoxylin and eosin stain). (A) Hematoxylin and eosin staining of metastatic lymph nodes obtained during primary breast cancer surgery. The tumor showed morphological similarities to the cervical recurrence. (B) Colposcopy‐directed biopsy of the uterine cervix showing round tumor cells infiltrating the cords and small nests. (C) The resected hysterectomy specimen exhibits tumor cells that infiltrate the cords, small nests, and occasional tubules, consistent with metastatic invasive ductal carcinoma.

## 3 Discussion

We encountered an extremely rare case of breast cancer metastasis to the uterine cervix occurring 30 years after the primary surgery. The following points address the diagnostic challenges and the clinical significance of this case.

### 3.1 Metastatic Route and Histological Subtype

Although ILC is less common than IDC, it is known to metastasize to gynecological organs and the gastrointestinal tract, potentially via peritoneal seeding or retroperitoneal spread [7, 8]. The reported rate of metastasis to gynecological organs is approximately 4.5% for ILC versus only 0.8% for IDC [9]. Conversely, IDC typically spreads hematogenously to the lungs, bone, or liver and rarely metabolizes to the uterine cervix [4]. Even among gynecological metastases, the distribution is markedly uneven: the ovary is the most common site (approximately 76%), followed by the vagina (approximately 13%) and the uterine corpus (approximately 8%) [8], whereas the cervix is among the rarest site, accounting for approximately 2% of such metastases [1]. The cervix is considered a rare site of metastasis because of its small size, dense fibromuscular tissue, limited blood supply, and centrifugal lymphatic drainage [10]. In the present case, the tumor was histologically an IDC that formed a localized mass in the cervix without ovarian involvement. Given the patient's history of mediastinal lymph node recurrence, retrograde lymphatic spread represents a plausible mechanism.

### 3.2 Tumor Dormancy and Ultra‐Late Recurrence

The concept of tumor dormancy explains how disseminated tumor cells can remain quiescent for extended periods before reactivating [11]. Recurrences may occur very late; one study reported a 16.6% cumulative incidence of breast cancer recurrence between 10 and 32 years after diagnosis [5], and hormone receptor‐positive breast cancers, in particular, carry a persistent risk of recurrence exceeding 20 years [6]. In the present case, the interval from the primary breast surgery to cervical metastasis was 30 years. To contextualize this unusually long disease‐free interval, we reviewed previously reported cases of breast cancer metastasizing to the uterine cervix. Among all reported cases with a quantifiable interval (*n* = 28), the median interval from the primary breast cancer to cervical metastasis was approximately 4 years (range, 0.25–20 years). When stratified by histological subtype, the interval tended to be longer for ILC (median, 4 years; range, 2–15 years; *n* = 15) than for IDC (median, 2 years; range, 0.25–20 years; *n* = 9). Although IDC generally presented earlier, the longest previously reported interval for this subtype is 20 years [3]. The present case of IDC recurring after 30 years exceeds the maximum interval reported for either histological subtype to date (Table S1 summarizes cases with an interval ≥ 10 years).

### 3.3 Cytological Features

Haji et al. reported that metastatic tumors in uterine cervical cytology often lack tumor diathesis [2]. Primary invasive cervical adenocarcinomas typically exhibit feathering, pseudo‐stratification, and tumor diathesis [12]. In the present case, the background was clean, and the cells appeared as isolated cells or scattered clusters with ICL features atypical of primary cervical adenocarcinoma. These cytological characteristics served as early indications of the metastatic nature of the lesion.

### 3.4 Utility of Immunohistochemistry

Differentiating metastatic breast cancer from primary cervical adenocarcinoma is crucial. As morphological distinction is often challenging, an immunohistochemical panel including both breast markers (GATA3 and GCDFP‐15) and Müllerian markers (PAX8) is recommended. GATA3 is regarded as one of the most sensitive markers for breast cancer. Conversely, PAX8 is highly sensitive to carcinomas of the Müllerian tract but is consistently negative in breast cancer [13]. In the present case, the GATA3(+)/PAX8(−) profile was definitive for breast cancer. Although p16 was focally positive, this finding does not exclude breast cancer, whereas diffuse block positivity is characteristic of high‐risk human papillomavirus‐associated cervical adenocarcinoma [12].

### 3.5 Biomarker Discordance and Therapeutic Implications

In the present case, the primary and metastatic tumors were assessed using immunohistochemistry (ER, PgR, HER2, and others), but comprehensive molecular profiling, including next‐generation sequencing, was not performed. In breast cancer, receptor conversion—a change in ER, PgR, or HER2 status between the primary tumor and metastatic sites—is well recognized and can affect treatment decisions and prognosis [14]. Kutasovic et al. reported that metastases to gynecological organs frequently arise from luminal A‐like primary tumors and that, during metastatic progression, ER expression is generally maintained and the ER cofactor FOXA1 is even increased, whereas PgR, androgen receptor, and GATA3 are heterogeneously downregulated, indicating persistence of hormonal signaling [15]. Consistent with these findings, in previously reported cases of cervical metastasis occurring ten or more years after the primary breast cancer, recurrent lesions almost uniformly retained ER/PgR positivity when assessed (Table S1). This observation aligns with the recognized propensity of hormone receptor‐positive breast cancer to relapse after prolonged latency [5, 6]. In contrast, in the present case, after an approximately 30‐year disease course involving prolonged endocrine therapy (tamoxifen) and repeated systemic treatment, the tumor evolved from an ER‐positive/PgR‐positive luminal phenotype to an HER2‐positive phenotype with complete loss of ER and PgR, diverging from the pattern reported previously and the sustained hormonal signaling phenotype. This makes the receptor conversion in our case a particularly unusual event. Notably, identification of HER2 overexpression on repeat biopsy prompted the initiation of targeted anti‐HER2 therapy, illustrating the practical value of biopsy and biomarker reassessment even in very late metastatic recurrences.

We herein report a case of uterine cervical metastasis from an IDC that occurred 30 years after surgery for primary breast cancer. Although diagnosis was challenging owing to the exceptional latency, it was achieved through careful cytological evaluation and specific immunohistochemical studies. This case highlights the critical role of long‐term gynecological surveillance in breast cancer survivors. Gynecologists should maintain a high index of suspicion for metastasis when encountering abnormal cervical findings and cytology, regardless of the disease‐free interval.

## Author Contributions

**Taro Miyake:** conceptualization, investigation, writing – original draft, writing – review and editing, methodology, project administration, data curation, visualization, formal analysis. **Hidenori Sasa:** conceptualization, writing – review and editing, validation, supervision. **Tsubasa Ito:** investigation, resources. **Takanori Watanabe:** validation, writing – review and editing. **Takako Sato:** validation, writing – review and editing. **Sho Ogata:** validation, writing – review and editing, supervision. **Masashi Takano:** writing – review and editing, supervision.

## Disclosure

An earlier version of this work was presented at the 78th Annual Congress of the Japan Society of Obstetrics and Gynecology, held on May 17, 2026.

## Ethics Statement

The Institutional Review Board of the National Defense Medical College Hospital determined that ethical approval was not required for this single‐patient case report, in accordance with institutional policy.

## Consent

Informed consent was received from the patient.

## Conflicts of Interest

The authors declare no conflicts of interest.

## Supporting information


**Table S1:** Long‐interval (≥ 10 years) breast cancer metastases to the uterine cervix: histology, interval, and receptor status cervical.

## Data Availability

The data supporting the findings of this study are not publicly available because they consist of identifiable clinical, radiological, and histopathological information derived from a single patient. Public sharing of these data would compromise patient privacy and breach the confidentiality conditions under which consent was obtained and under which our institution's ethical policy operates. De‐identified data that do not permit identification of the patient are available from the corresponding author (T.M.) upon reasonable request, subject to approval by the Institutional Review Board of the National Defense Medical College Hospital and to a data‐sharing agreement that safeguards patient confidentiality.
