# Supplementary material for: Metastasis of Invasive Ductal Carcinoma of the Breast to the Uterine Cervix 30 Years Post‐Mastectomy: A Case Report
Source: J Obstet Gynaecol Res. 2026 Sep 28;52(10):e70509. doi: 10.1111/jog.70509 (PMC13620436; doi:10.1111/jog.70509)
Supplement: Supplementary file 1 [file JOG-52-0-s001.doc]

**Supporting Information**

*Metastasis of Invasive Ductal Carcinoma of the Breast to the Uterine Cervix 30 Years Post-mastectomy: A Case Report*

**Supplementary Table S1. Long-interval (≥10 years) breast cancer metastases to the uterine cervix: histology, interval, and receptor status cervical**

| **Reference (year)** | **Histology** | **Interval (years)** | **Primary tumor ER / PgR / HER2** | **Cervical recurrent lesion ER / PgR / HER2** |
| --- | --- | --- | --- | --- |
| Cochrane et al. (2020) | IDC | 10 | ER +  PR +  HER2 + | ER+  PR+  HER2 NR |
| Pellecchia et al. (2026) | ILC | 10 | ER +  PR +  HER2 − | ER+  PR+  HER2 1+ (low) |
| Ogino et al. (2003) | ILC | 10.7 | NR | NR |
| Manci et al. (2008) | ILC | 11 | ER +  PR +  HER2 − | ER +  PR +  HER2 − |
| Waks et al. (2015) | ILC | 15 | ER +  PR +  HER2 NR | ER +  PR +  HER2 − |
| Dye et al. (2024) | IDC | >20 | NR | ER +  PR+  HER2 NR |
| Present case | IDC | 30 | ER +  PgR +  HER2 − | ER −  PgR −  HER2 + |

Interval, time from the primary breast cancer diagnosis/surgery to cervical metastasis. In all previously reported cases in which receptor status could be assessed, the recurrent lesion retained ER/PgR positivity; the present case is distinctive in showing loss of ER/PgR and gain of HER2. IDC, invasive ductal carcinoma; ILC, invasive lobular carcinoma; ER, estrogen receptor; PgR/PR, progesterone receptor; HER2, human epidermal growth factor receptor 2; NR, not reported

**Supplementary References**

1. Cochrane E, Kim S, Kudelka A, Burke W. Invasive ductal breast carcinoma metastasis to the cervix: a case review and clinical correlation. Gynecol Oncol Rep 2020; 33: 100616.

2. Dye A, Jhaveri V, Ozdemir S, Alkhasawneh A, Hew K. Recurrent invasive ductal carcinoma of the breast with metastasis to the uterine cervix: a case report. Case Rep Womens Health 2024; 42: e00607.

3. Manci N, Marchetti C, Esposito F, et al. Late breast cancer recurrence to the uterine cervix with a review of the literature. Int J Gynecol Pathol 2008; 27: 113-117.

4. Ogino A, Nomizu T, Gonnda K, et al. A case of breast cancer metastasizing to cervix after resection of pancreatic metastasis. Breast Cancer 2003; 10: 284-288.

5. Pellecchia G, Restaino S, Alfarè Lovo M, et al. Late cervical recurrence of invasive lobular carcinoma ten years after primary breast cancer: a case report and review of the literature. Healthcare (Basel) 2026; 14: 201.

6. Waks AG, Lennon J, Yadav BS, et al. Metastasis to the cervix uteri 15 years after treatment of lobular carcinoma of the breast. Semin Oncol 2015; 42: e81-e94.
